# A modified method for reconstruction of posterior tibial tendon after resection of juvenile painful type II accessory navicular

**DOI:** 10.1186/s13018-023-04383-3

**Published:** 2023-11-29

**Authors:** Haoli Gong, Yuyin Xie, Zhenqi Song, Zhongwen Tang, Jie Wen, Sheng Xiao

**Affiliations:** 1 Department of Pediatric Orthopedics, Hunan Provincial People’s Hospital, The First Affiliated Hospital of Hunan Normal University, Changsha, 410005 China; 2 Department of Anatomy, Hunan Normal University School of Medicine, Changsha, 410013 Hunan China

**Keywords:** Juvenile type II accessory navicular, Posterior tibial tendon reconstruction, *AOFAS*-*AH*, Meary angle, Pitch angle

## Abstract

**Background:**

The surgical treatment of accessory navicular (AN) is divided into simple resection of AN and Kidner surgery used to reconstruct posterior tibial tendon (PTT) after AN resection. However, both of these procedures have certain disadvantages. Herein, we proposed a modified method to reconstruct PTT and compared the short-term clinical effect of our method with the modified Kidner procedure.

**Methods:**

We collected data from 23 adolescent children with painful type II AN treated in our department between January 2015 and June 2020. The American Orthopedic Foot and Ankle Society Ankle-Hind foot (AOFAS-AH) Scores, the Meary Angle, and Pitch Angle of the lateral weight-bearing plain radiographs status were recorded before and after the operation to evaluate the treatment outcomes.

**Results:**

In the modified Kidner surgery (MK) group, the median *AOFAS*-*AH* increased from 61 (59–68) to 87 (83–91) (*P* < 0.05); the Pitch angle of the lateral weight-bearing plain radiographs increased from 13.0 (8–18) to 17.4 (14–22), and the Meary angle decreased from 18.3 (14–24) to 14.2 (8–20) (*P* < 0.05). In the PTT preservation folded suture (FS) group, the median AOFAS-AH increased from 61 (59–68) to 87 (85–91) (*P* < 0.05); the Pitch angle of the lateral weight-bearing plain radiographs increased from 12.3 (7–18) to 18.4 (15–26), and the Meary angle decreased from 17.8 (13–23) to 5.7 (3–8) (*P* < 0.05). There was no significant difference in *AOFAS*-*AH* postoperative scores between the FS group and MK group; however, the improvement on Pitch and Meary angle of the lateral weight-bearing plain radiographs was significantly better in the FS group than in MK group (*P* < 0.05).

**Conclusions:**

For painful type II AN in juvenile patients, the insertion-preserving folding suture procedure had similar short-term results on AOFAS-AH scores but greater improvement in the Meary angle and the Pitch Angle than the modified Kidner method.

Level of Evidence: III

## Introduction

The accessory navicular bone is the most common accessory bone in the foot, found in 4–21% of patients [1, 2]. The incidence rates do not significantly differ among boys and girls; however, from adolescence onwards, the incidence is higher in women than men. The age of onset is also younger in women, which may be related to skeletal maturity that women achieve earlier than men [3, 4]. The disease can be bilateral or unilateral [5, 6] and is currently considered as an autosomal dominant inheritance [7].

Accessory navicular can be divided into 3 types according to radiology results[8], i.e., Type I: the AN is round or oval, with a clear boundary, not connected with the navicular bone and generally asymptomatic; Type II: the tuberous fibrous cartilage plates are separated between the navicular and the AN. Type IIA is connected to the talus process and mainly affects tensile force, while Type IIB is more localized to the metatarsal side and mainly affects shear force. The two subtypes can be identified by 45° valgus oblique radiography [4].Type III is a bone bridge that connects the accessory navicular bone with the navicular bone. According to existing literature, most painful AN is caused by type II AN [9, 10]. There is no real joint between the AN and navicular, only connective tissue or supporting tissues such as cartilage-like and fibro-cartilage. The damage of the fibro-cartilage plate under the combined force of tensile and shear and the damage to the pseudo-articular joint may be the main reason for pain in type II AN [11].

The treatment for AN can be divided into conservative and surgical treatment, with the former including the insole, casts [12]. Nonetheless, conservative treatment is often ineffective for Type II AN patients [13]. Jegal et al*.*[14] used the conservative treatment on 29 athletes and 50 ordinary patients, finding it effective in 34% of ordinary patients and only 6.9% of athletes. Therefore, surgical intervention is often indicated when conservative treatment fails. Surgical treatment includes simple AN resection [13], Kidner procedure [15], and modified Kidner procedure [16], with different methods being used for the reconstruction of the PTT. However, the Kidner procedure involves separating the PTT with small bone fragments, removing the AN and using chromic suture to reconstruct the free posterior tibial tendon with small bone fragments on the medial plantar surface of the navicular tubercle to achieve the reconstruction, which may increase the risk of damage to the medial support system of the foot.

Based on the pathological characteristics of children with type II AN, we proposed a new method to reconstruct the PTT, which involves reconstructing the PTT by folding the tendon with its retained insertion and suturing it to the navicular using a suture anchor after the accessory navicular(AN) resection. The short-term clinical effects of our method were compared with the effect of the modified Kidner procedure.

## Methods

Patients with painful type II AN who treated with modified Kidner surgery and PTT preservation folded suture surgery in our department between January 2015 and June 2020 were included in this study. All the children were divided into modified Kidner surgery group (MK group) and preservation folded suture group (FS group) according to different surgical methods. Inclusion criteria were the following: (1) those with history, physical examination, and X-ray confirmed diagnosis of type II AN; (2) no previous foot surgery history; (3) the presence of foot pain, which could not be relieved by conservative treatment.

Exclusion criteria were: (1) foot deformity with tarsal syndesmosis, vertical talus and other bone malformations; (2) other types of AN; (3) patient received other types of surgery.

### Surgical methods

**Fig. 1:**
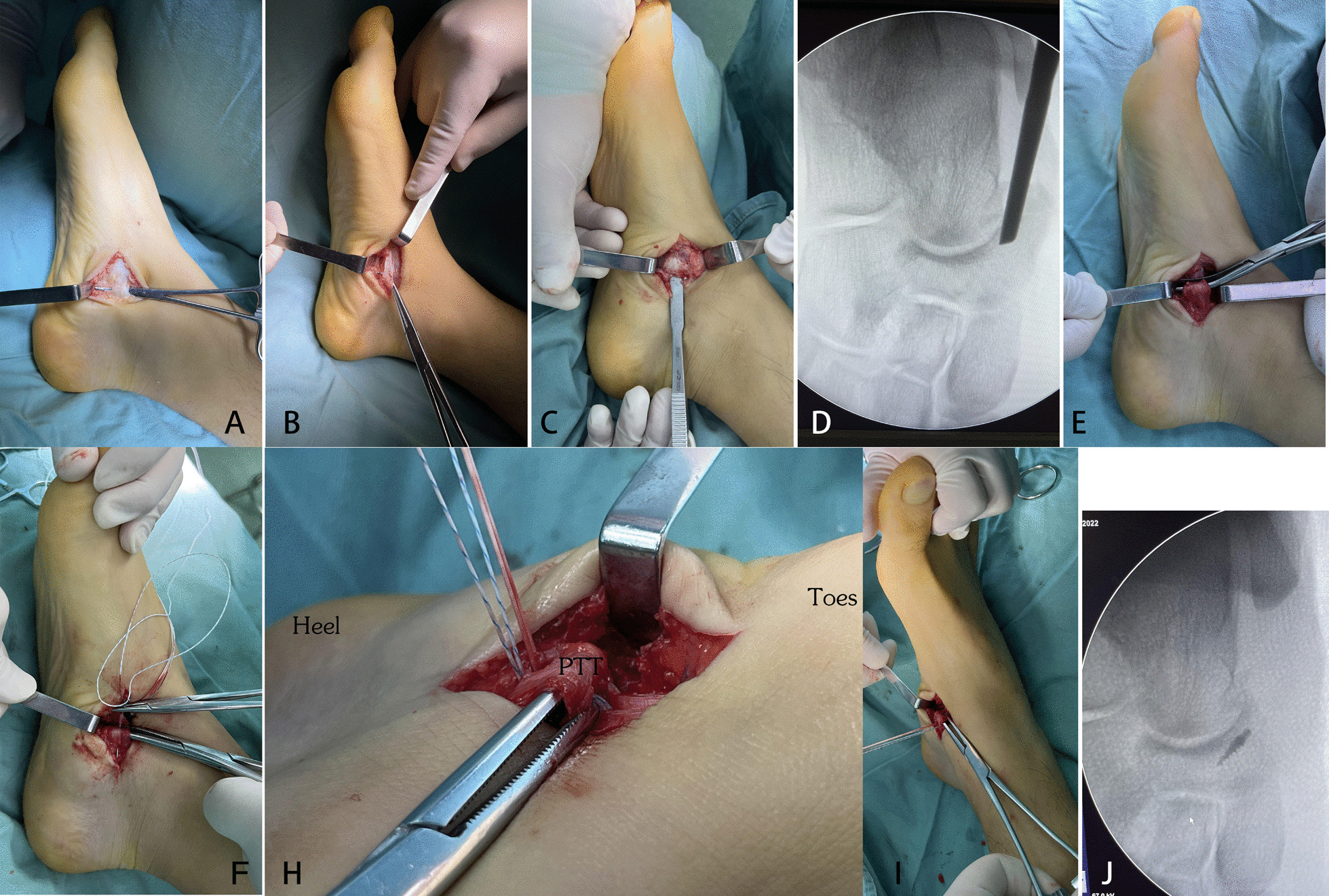
Intraoperative procedure of posterior tibial tendon insertion preserved in folded suture surgery. **A** Incision. **B** Explosion of PTT. **C** AN resection. **D** Intraoperative fluoroscopy. **E** PTT fold method. **F** PTT suture method. **H** PTT folded and sutured. **I** PTT folded and sutured (different visual angle). **J** Anchor position in fluoroscopy

**Fig. 2:**
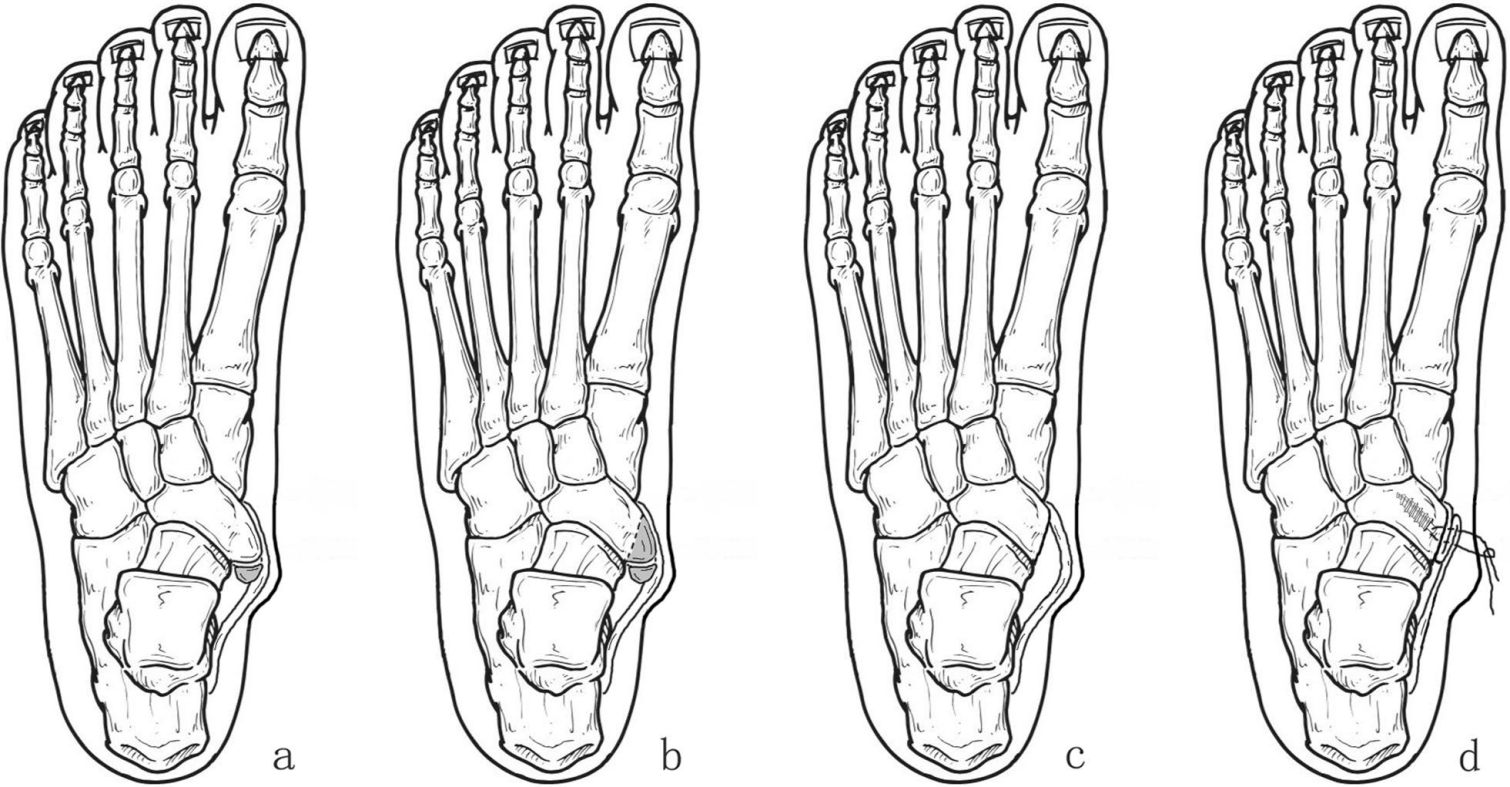
Illustration of posterior tibial tendon insertion preserved and folded suture surgery (AP view). **a** AN before operation. **b** Resection range in operation. **c** PTT loose after AN resection. **d** PTT folded and sutured after anchor insertion. Please note the folded distance can be adjust according to different patients

Both operations were performed under general anesthesia, and the medial approach of the accessory navicular was used in both groups. Patient was lying in a supine position, and a 2–3 cm incision was made at the prominence of the accessory navicular bone on the medial foot. The skin and superficial fascia were incised to expose the posterior tibial tendon (Fig. 1A), after which the posterior tibial tendon was peeled off from the outer layer of accessory navicular and proximal navicular (Fig. 1B). During the operation, the continuity of the posterior tibial tendon was kept intact, and the insertion of the posterior tibial tendon was preserved. The retractor was used to pull the posterior tibial tendon aside to expose the accessory navicular bone (Fig. 1C). The AN was excised under fluoroscopy (Fig. 1D), and the residual navicular bone was polished, after which a suture anchor was placed in the middle of the navicular (Fig. 2).

In patients treated with modified Kidner surgery(MK group), the posterior tibial tendon was detached from its insertion point and tightened and attached to the navicular by the anchor placed after the foot was maintained in a clubfoot position. The incision was sutured, and a cast was used to keep the foot in the clubfoot position.

**Fig. 3:**
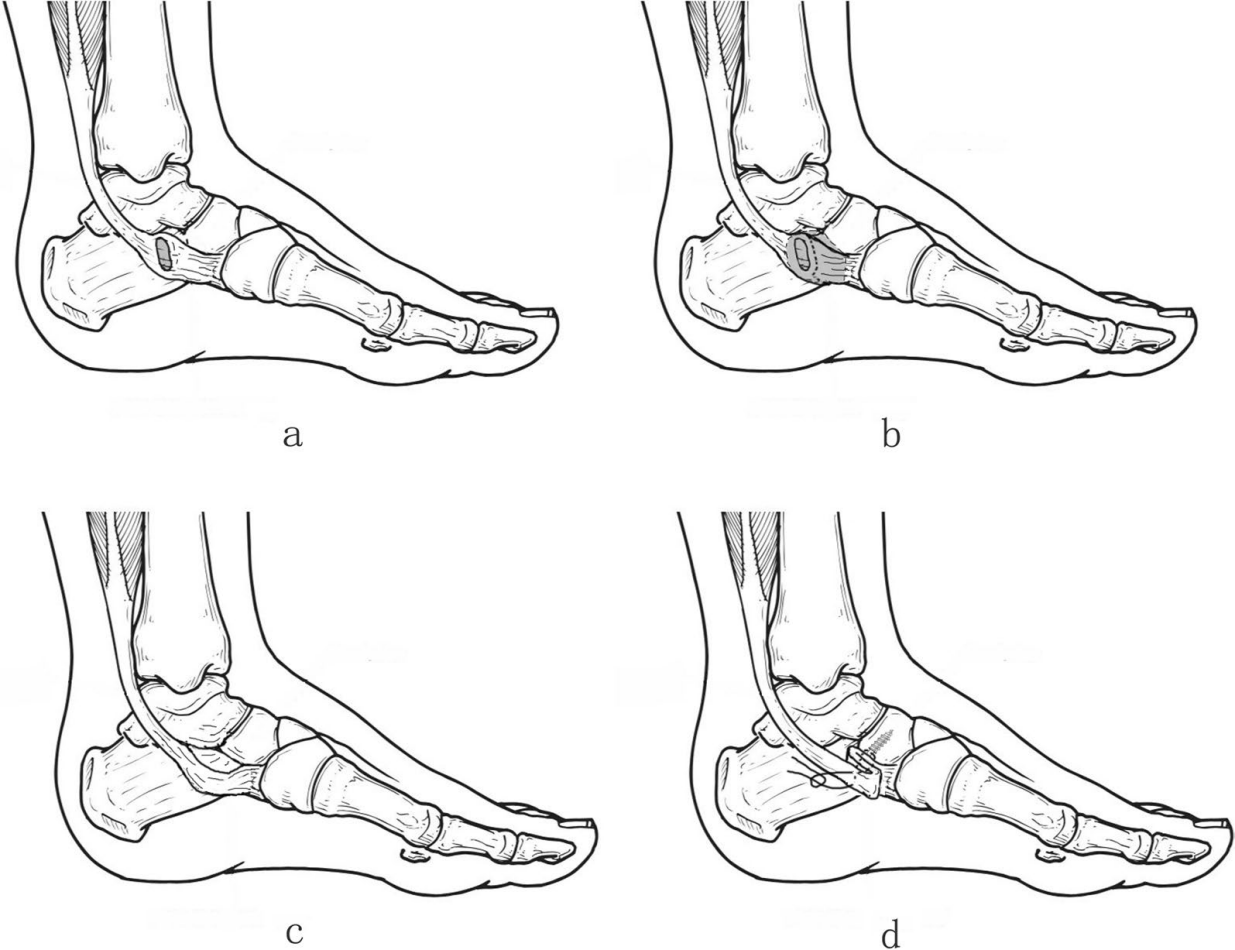
Illustration of posterior tibial tendon insertion preserved and folded suture surgery (Lateral view). **a** AN before operation. **b** Resection range in operation. **c** PTT loose after AN resection. **d** PTT folded and sutured after anchor insertion. Please note the folded distance can be adjust according to different patients

In patients treated with posterior tibial tendon folded suture and insertion preserved (FS group), the insertion of PTT was preserved (Fig. 3), and PTT was folded sutured using the suture of the anchor (Fig. 1E–H) after the foot was maintained in a neutral position and PTT maintained no tension. Next, PTT was sutured with the surrounding soft tissue for reinforcement; the incision was sutured, and a cast was used to keep the foot in a neutral position.

### Postoperative treatment

All children were immobilized with long-leg casts for 4 weeks after the operation. If the children were asymptomatic, the anchors with thread were not removed and were left in the navicular bone. After 4 weeks, the plaster was removed, and the patient was instructed to do the rehabilitation for 2 weeks. Subsequently, the patient began walking wearing foot arch support insoles for at least 1 month.

### Evaluated parameters

After discharge, outpatient reexamination was conducted, once every 3 months and once every six months after 1 year. A fixed physician recorded the *AOFAS-AH* function Score at each outpatient follow-up visit to evaluate the clinical outcomes. X-ray examinations were performed every 3 months. The Meary angle and the Pitch angle in the X-ray of the last follow-up were measured and compared to the preoperative X-ray.

### Statistical analysis

SPSS 21.0 software was used for statistical analysis. The measurement data were expressed in median value and range (min, max). The preoperative and postoperative scores and imaging indicators were compared by the nonparametric test of two related samples, and the scores and imaging indicators between the two groups were compared by the nonparametric test of two independent samples. The test level α value was 0.05 on both sides.

## Results

A total of 12 children (19 feet), 5 boys and 7 girls, aged 9–14 years, mean 11.8 years old were treated with modified Kidner surgery (MK group). There were 7 cases of bilateral surgery and a mean follow-up time of 17.7 ± 2.6 months (14–22). A total of 11 children (18 feet), 5 boys and 6 girls, aged 10–14 years, with a mean of 12.2 years old, were treated with posterior tibial tendon folded suture and preserved insertion (FS group). Seven patients underwent bilateral surgery; their mean follow-up time was 17.1 ± 3. 2 months (12–22).

**Fig. 4:**
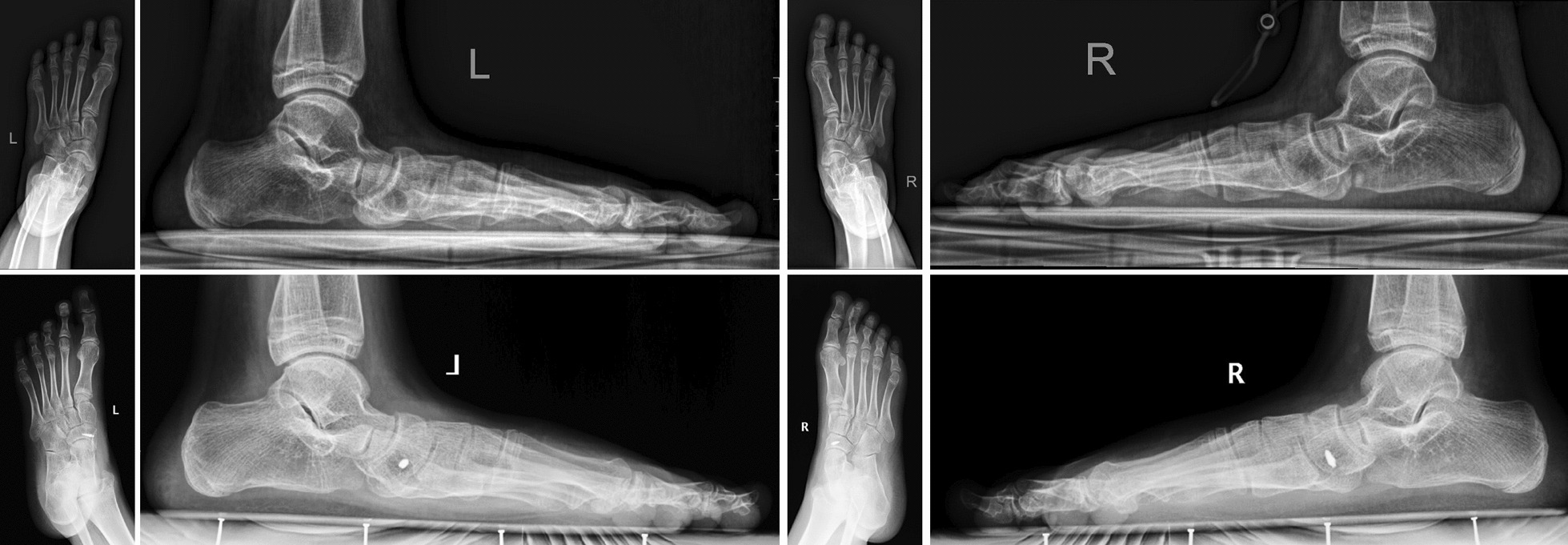
Case in FS group, case number 21, male, bilateral AN, Pitch angle Pre-op: both 9 degree, Pitch angle in Last FU: lef 15 degree, Meary angle Pre-op: both 22 degree, Meary angle in Last FU: left 6 degree, right 3 degree

All patients experienced significant improvement in postoperative foot pain symptoms and alignment (Table 1). In the MK group, the median preoperative AOFAS-AH score was 61 points (59–68), and the median postoperative score was 87 (83–91); the differences were statistically significant (*P* < 0.05). The Pitch Angle of postoperatively loaded foot lateral radiographs increased from 13.0 degrees (8–18) to 17.4 degrees (14–22), and the actual delta values of the Pitch Angle from pre-operation to last follow-up were 4 (2–8). The Meary angle decreased from 18.3 degrees (14–24) to 14.2 degrees (8–20); the observed difference was statistically significant (*P* < 0.05), and the actual delta values of the Meary Angle from pre-operation to the last follow-up were 4 (0–11). A typical case is illustrated in Fig. 4.

**Table 1:** General information of selected adolescents

No	Gender	Age	Bilateral/Unilateral	Group	FU (M)	Side	AOFAS		Pitch angle		Meary angle	
							Pre-op	Last FU	Pre-op	Last FU	Pre-op	Last FU
1	M	11	B	MK	16	L	64	85	14	17	18	16
						R	61	87	15	18	17	16
2	F	13	U	FS	15	L	59	87	15	20	20	6
3	F	10	B	FS	12	L	64	88	10	16	19	5
						R	64	87	11	16	18	5
4	M	9	B	MK	17	L	64	84	10	14	20	19
						R	64	85	11	15	20	20
5	F	12	B	MK	14	L	59	83	9	15	19	18
						R	61	85	8	16	21	19
6	M	10	U	FS	20	L	61	85	13	20	14	7
7	M	14	B	FS	18	L	64	88	9	15	16	5
						R	61	87	10	16	16	3
8	M	12	B	FS	22	L	64	91	8	15	18	8
						R	61	87	7	15	20	6
9	F	13	U	MK	22	L	61	91	18	22	18	16
10	F	11	B	MK	20	L	61	87	15	21	16	12
						R	64	88	15	19	17	10
11	F	11	B	FS	14	L	59	87	18	22	14	5
						R	59	85	18	23	13	4
12	M	14	U	MK	18	R	61	85	12	16	15	15
13	F	12	U	MK	16	L	59	87	14	18	19	14
14	F	13	B	FS	17	L	64	88	12	20	19	5
						R	61	85	13	19	20	7
15	F	14	U	FS	20	R	64	91	11	15	23	5
16	F	12	B	MK	15	L	64	85	9	13	19	17
						R	64	87	8	13	19	15
17	M	13	U	MK	20	L	68	91	10	15	20	9
18	F	11	B	MK	21	L	61	87	16	18	22	14
						R	61	85	16	20	24	13
19	F	13	B	FS	13	L	61	87	17	25	15	6
						R	61	85	18	26	16	8
20	M	12	U	FS	18	L	68	85	13	18	16	8
21	M	12	B	FS	19	L	64	87	9	15	22	6
						R	61	88	9	15	22	3
22	M	12	U	MK	15	R	64	88	12	18	15	10
23	F	11	B	MK	18	L	59	87	18	22	14	8
						R	61	85	17	21	14	9

**Fig. 5:**
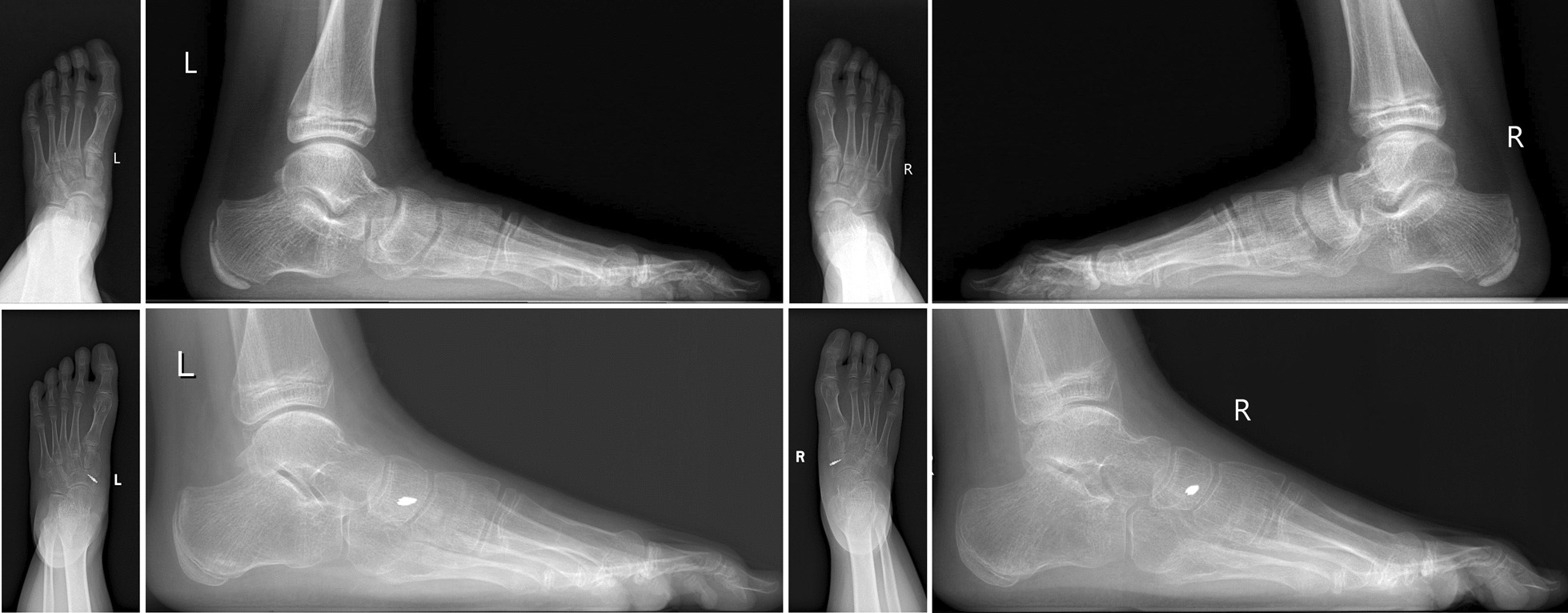
A typical Case in FS group. Case number 21, male, bilateral AN, Pitch angle Pre-op: both 9 degree, Pitch angle in Last FU: left 15 degree, Meary angle Pre-op: both 22 degree, Meary angle in Last FU: left 6 degree, right 3 degree

In the FS group, the median preoperative AOFAS-AH score was 61 points (59–68), and the median postoperative score was 87 (85–91); the differences were statistically significant (*P* < 0.05). The Pitch angle of postoperatively loaded foot lateral radiographs increased from 12.3 degrees (7–18) to 18.4 degrees (15–26); the actual delta values of the Pitch angle from pre-operation to the last follow-up were 6 (4–8). The Meary angle significantly decreased from 17.8 degrees (13–23) to 5.7 degrees (3–8) (*P* < 0.05); the actual delta values of the Meary angle from pre-operation to the last follow-up were 13 (7–19). There was no significant postoperative difference in AOFAS-AH scores between the FS group and MK group; however, the actual delta values on the Pitch and Meary angles of the lateral weight-bearing plain radiographs showed greater improvement in FS group than in the MK group; the observed difference was statistically significant (*P* < 0.05) (Table 2). A typical case is illustrated in Fig. 5.

**Table 2:** Comparison of two groups

	FS group	MK group
Total		
Patients	11	12
Feet	18	19
Gender		
Male	5	5
Female	6	7
Side		
Left	10	10
Right	8	9
Age (yo)	12.2	11.8
FU (m)	17.1	17.7
AOFAS-AH scores		
Pre-OP	61	61
Last FU	87	87
*δ* value	25	24
Pitch angle		
Pre-OP	12.3	13.0
Last FU	18.4	17.4
δ value	6.0	4.0*
Meary angle		
Pre-OP	17.8	18.3
Last FU	5.7	14.2
*δ* value	13.0	11.0*
Complications		
Infections	0	0
Pain	1	1

^*^P < 0.05

All patients were followed up for 12–22 months, with an average follow-up time of 17.4 ± 2.9 months. There were no incision problems or vascular and nerve injuries during the follow-up in any patients. Each group had 1 case of local pain, both received bilateral surgery and the pain comes from the right foot. In the FS group, 1 patient experienced pain in the suture area of the posterior tibial tendon caused by increased tension. However, after wearing footpads for 6 months, no pain or mobility disorder symptoms appeared, so a second operation was not required. In MK group 1 patient experienced pain in the AN resection area, which was relieved after wearing footpads for 6 months. Nonetheless, the patient underwent a second operation by inserting a subtalar joint stabilizer, and the pain disappeared after surgery. The follow-up of all patients is still ongoing.

## Discussion

In 1925, Geist et al*.* [18] proposed the simple resection of the accessory navicular bone. He reported that the simple resection of the accessory navicular bone and trimming the residual bony process did not require complete cutting of the posterior tibial tendon(PTT) insertion and thus, could preserve and maintain the continuity and function of PTT. He emphasized the importance of partial PTT freeing and repairing the tendon damaged by stripping. In 1929, Kidner et al*.*reported using AN resection and PTT reconstruction for the treatment of AN patients in order to maintain the medial longitudinal arch of the foot and the dynamic basis of flexion and inversion of the toes. He suggested separating PTT with small bone fragments, removing the AN and suturing with chromic suture to reconstruct the free PTT with small bone fragments on the medial plantar surface of the navicular tubercle so as to achieve tendon reconstruction and obtain a good therapeutic effect [15]. Macnicol et al*.* [19] compared the clinical effect of Kidner surgery and simple excision in treating painful AN. He performed Kidner surgery on 26 AN patients with severe flat feet and used simple excision in 21 patients with painful AN. The follow-up results showed no significant difference in foot pain relief between the two groups after the operation (*P* > 0.05). In their study, Cha et al*.* [20] performed Kidner surgery and simple resection on 50 AN children, respectively, and 25 cases in each group were followed up for 3 years. The clinical results were good in both groups, and the re-alignment of the foot arch was similar.

However, both procedures have disadvantages. The simple resection of the AN cannot achieve reconstruction and suture fixation of the loose PTT but only surgical removal of the AN. The pain was reported to worsen in patients with severe flat foot deformity, who complained of unrelieved symptoms after surgery, while even progressive aggravation and recurrence were possible [21]. Kidner surgery may cause damage to the medial support system of the foot. If a firm insertion of the PTT cannot be achieved, tendon relaxation is likely to occur. Repeated friction of PTT causes aseptic inflammation around the insertion and local scar hyperplasia, thus preventing pain relief [22]. Choi et al*.* [23] reported recurrent pain in 9 patients after Kidner surgery. They argued that after Kidner surgery was performed in patients with flat feet, the tension in the attachment point of the PTT increased, resulting in tendon degeneration and recurrent foot pain.

Along with the development and progress of orthopedic implants, many scholars modified the Kidner procedure by inserting suture anchors into the navicular and then reconstructing PTT using the suture anchors. Dawson conducted a comparative study of 13 patients, 7 of whom were treated with suture anchors, finding that the use of anchors could shorten the postoperative recovery time of walking [23]. Kakihana et al*.* performed PTT reconstruction with thread anchors in 15 adolescent patients who were followed up for at least 1 year, finding significant relief of symptoms during short and mid-term follow-up with satisfactory radiology results [25]. Many studies have shown that using suture anchors could make operations simple, minimally invasive, reliable, and safe. Moreover, it could help to avoid secondary surgery. The suture anchor has excellent tissue compatibility while in contact with bone tissue. The built-in strong wear-resistant and tension-resistant polyester fiber suture can stably and firmly weave and suture the PTT, thus providing stability and benefiting PTT fused to the navicular. Therefore, we sutured and fixed PTT with suture anchors in both groups in the present study.

The relationship between AN and flatfoot has always been the focus of interest among researchers. Kidner argued that the abnormal anatomy of PTT weakens the maintenance force on the medial longitudinal arch of the foot, resulting in flatfoot. At the same time, the alignment of PTT changes due to the protruding AN, which may cause impingement from AN, resulting in painful bursitis, PTT tendonitis, and eventually flatfoot [15]. Kiter et al. pointed out that the PTT inserts directly on the AN could weaken the stability of the talonavicular ligament. As PTT has no supinator function without its distal attachments, the gastrocnemius-soleus complex acts at the talonavicular joint, making the passive structures of the longitudinal arc collapse and causing flatfoot [26]. Bernaerts et al*.* suggested that the presence of type II and type III AN could easily lead to posterior tibial tendinopathy because, in the presence of type II and III AN, most of the posterior tibial tendons are inserted into the AN, shortening the insertion point of the PTT, reducing the leverage of the ankle on the posterior tibial tendon and resulting in increased tendon stress [27]. In the current study, 1 patient from the MK group had pain in the AN resection area. After wearing footpads for 6 months, the pain was slightly relieved. The patient underwent a subtalar joint stabilizer (HyProCure) procedure, and the pain disappeared after the second surgery, which indicated that the pain of AN is different from flatfoot.

Some scholars reported that AN does not cause flatfoot and that AN and flatfoot are accidental phenomena. Sullivan et al*.* suggested that the presence of the AN does not participate in developing flatfoot [28]. Micheli and colleagues argued that the AN does not cause flatfoot. On the contrary, the excessive tension and traction of the PTT during the ossification of the navicular induce the formation of AN [29].

Senses et al*.* [30] performed AN simple resection on 8 patients with flat foot deformity and restored PTT's continuity, finding that PTT had insertion points on both AN and navicular. After resected the AN, they sutured the main trunk of PTT to the navicular. During the 2-year follow-up, all 8 patients could complete the single-foot heel raise. There was no change in the lateral Meary angle of 6 patients, while the angle was only reduced by 2° in 2 patients. They suggested that this procedure did not significantly improve the collapse of the longitudinal arch of the foot, thus providing evidence that the AN was not associated with flatfoot. In the present study, we stripped the PTT from the outer layer of the accessory navicular cartilage surface in all 23 cases, which certified that PTT has insertion points both on AN and navicular. Our results also showed that the continuity of the PTT could be kept intact and does not need to be cut off. Therefore, we proposed to preserve PTT insertion and reconstruct the PTT by folding it twice and suturing it to the navicular. By using a double fold, the posterior tibial tendon was tightened, and the overlapping distance of the tendon could be determined intraoperatively according to the relaxation of PTT. This method retains the advantages of the Geist and Kidner procedures and helps to avoid the anchor displacement caused by the excessive concentration of stress points on the anchor after PTT amputation and reconstruction. The improvement of the AOFAS score after the operation suggested that our methods could effectively stop foot pain and restore foot function. The improvement in Meary angle and Pitch angle suggested that the alignment of the foot was better after PTT reconstruction.

During the follow-up period in the current study, there was only 1 patient in the FS group who experienced pain in the insertion area of the posterior tibial tendon caused by increased tension of the posterior tibial tendon in the right foot. No reoperation was performed for pain and movement disorder symptoms; the follow-up is still underway. It is possible that the maintained angle of the ankle joint plantar flexion and forefoot varus was excessive when we sutured the PTT, resulting in an over-shortening of the PTT, which caused tension increase. This highlights the critical importance of maintaining the position of the foot and ankle when folding PTT and suturing it to the navicular. PTT should be sutured to the medial wall of the navicular when the ankle joint is maintained in a neutral position. Unfortunately, we only had a short-term follow-up results. Yet, we plan to observe the clinical results further during mid-term and long-term follow-up, aiming to obtain more detailed data.

## Conclusion

After the resection of the juvenile type II AN, it was found that both methods used to manipulate the posterior tibial tendon, i.e., insertion-preserving folding suture and the modified Kidner procedure, could both achieve good clinical results. Also, the short-term results on AOFAS-AH function scores were similar. Comparing the actual delta values of the Meary angle and the Pitch Angle in lateral foot radiographs of weight-bearing state before and after surgery suggests that the insertion-preservation folding suture method can improve more in foot alignment than the modified Kidner method.

## Data Availability

The datasets used and/or analyzed during the current study are available from the corresponding author on reasonable request.
